# Immobilization Technologies in Probiotic Food Production

**DOI:** 10.1155/2013/716861

**Published:** 2013-10-28

**Authors:** Gregoria Mitropoulou, Viktor Nedovic, Arun Goyal, Yiannis Kourkoutas

**Affiliations:** ^1^Applied Microbiology and Molecular Biotechnology Research Group, Department of Molecular Biology & Genetics, Democritus University of Thrace, 68100 Alexandroupolis, Greece; ^2^Faculty of Agriculture, Department of Food Technology, University of Belgrade, Nemanjina 6, Zemun, 11081 Belgrade, Serbia; ^3^Department of Biotechnology, Indian Institute of Technology Guwahati, Guwahati, Assam 781039, India

## Abstract

Various supports and immobilization/encapsulation techniques have been proposed and tested for application in functional food production. In the present review, the use of probiotic microorganisms for the production of novel foods is discussed, while the benefits and criteria of using probiotic cultures are analyzed. Subsequently, immobilization/encapsulation applications in the food industry aiming at the prolongation of cell viability are described together with an evaluation of their potential future impact, which is also highlighted and assessed.

## 1. Introduction

Probiotics have rapidly gained interest in the area of self-care and complementary medicine under the general term “functional foods.” Modern consumers are increasingly interested in their personal health and particularly in foods which are capable of preventing and/or curing illness. Microbes have been used for years in food and alcoholic fermentations but only recently have undergone scientific scrutiny to examine their possible health benefits. 

The word “probiotic” comes from the Greek words “pro” and “biotic,” meaning “for the life.” The concept of “probiotics” appeared a long time ago. The Nobel laureate Elie Metchnikoff was the first microbiologist in the beginning of the 20th century who suggested that the longevity of Bulgarian peasants could be related to their large consumption of sour milk containing *Lactobacillus bulgaricus*. The most commonly used definition for probiotics comes from Fuller in 1989 defining that “probiotics are live microbial food supplements, which beneficially affect the host animal by improving its intestinal microbial balance” [1]. Salminen et al. [2] altered the term to “probiotics are microbial cell preparations or components of microbial cells that have a beneficial effect on the health and well-being of the host.” According to this definition, probiotics are not necessary to be viable, as nonviable forms have also been proved to provide health effects [3]. Today, the term “probiotic” refers to “live microorganisms which, administered in adequate amounts, confer a beneficial physiological effect on the host,” according to the Food and Agriculture Organization and World Health Organization [4]. 

A variety of microorganisms have been studied for potential probiotic effects. Most microbial strains with probiotic activity belong primarily to *Lactobacillus* and *Bifidobacterium* genera. However, the potential probiotic roles of other microbes are also under investigation.  Table 1 presents the most common microorganisms investigated for probiotic properties. 

Immobilization/encapsulation of probiotics is an exciting field of food technology that has emerged and developed rapidly in the past decade. The most excellent application of probiotic immobilization technology is the controlled and continuous delivery of cells in the gut. The potential benefit of this therapeutic strategy is to maintain greater cell viability despite the acidity of the stomach. In their viable state, probiotics exert a health benefit on the host. 

There is growing scientific evidence to support the concept that the maintenance of healthy gut microbiota may provide protection against gastrointestinal disorders, such as gastrointestinal infections and inflammatory bowel diseases [5–7]. The use of probiotic bacterial cultures may stimulate the growth of preferred microorganisms, crowd out potentially harmful bacteria, and reinforce the body's natural defence mechanisms [7]. Beneficial effects of probiotic consumption also include enhancement of bioavailable nutrients, reduction of symptoms of lactose intolerance, decrease of the occurrence of allergic symptoms in susceptible individuals, and reduced risk of certain cancers [5–7].  Table 2 summarizes the most important beneficial effects of probiotics on humans. However, the mechanisms by which probiotics exert their effects are largely unknown and may involve modification of gut pH, antagonism of pathogens through production of antimicrobial and antibacterial compounds, competition for pathogen receptor sites and for available nutrients and growth factors, stimulation of immunomodulatory cells, and production of lactase [8]. 

The overall objective of this review is to analyze and assess the data on immobilization technology of probiotic microorganisms for application in food production.

## 2. Criteria of a Culture to Be Used as Probiotic

Several aspects, including safety, functional and technological characteristics, have to be taken into consideration in the selection process of probiotic microorganisms. Many microorganisms could be considered as potential probiotics, but only a few are able to satisfy the necessary criteria.

Safety aspects include specifications such as origin (healthy human gastrointestinal tract), nonpathogenicity, nondigestive upsets, and nonantibiotic resistance characteristics.

Functional aspects include viability and persistence in the gastro-intestinal (GI) tract, surviving the digestive stresses [9], immunomodulation, and antagonistic and antimutagenic properties [10, 11].

Careful screening of probiotic strains for their technological suitability can also allow selection of strains with the best manufacturing and food technology characteristics. Moreover, they should not produce off-flavours [12]. 

An overview of the most significant criteria to define a probiotic microorganism is presented in Table 3.

### 2.1. Safety Criteria

It is essential that probiotics must be considered as “generally recognized as safe” (GRAS) organisms for human use according to the US Food and Drug Administration [13]. The congruent safety aspects include mainly human origin of strains in order to exclude negative characteristics as harmful effects, pathogenecity, digestive upsets, and antibiotic resistance. In particular:

1. it is highly recommended that strains used for products addressed to humans should be of human origin. Additionally, a probiotic strain is expected to function better in a similar environment from where it was originally isolated (e.g., human GI-tract). Generally, probiotics should be isolated from healthy human GI tract. It is also considered that the safety criteria depend on our experience in food fermentations;
2. there should be no association with disease. Most intestinal microorganisms are not considered pathogenic in healthy individuals, but some intestinal bacteria are potentially pathogenic. Their growth and metabolism are influenced by the normal immune system in the digestive tract. The pathogenic microbes can potentially cause an infection even in a healthy host;
3. metabolic activity in the food matrix and in the intestine following consumption is an important safety criterion. For example, although tolerance of bile salts is an essential criterion for the selection of potential probiotic strains, microbial bile salts hydrolase activity has been mooted to be potentially detrimental to the human host, and thus it is yet not completely clear whether it is in fact a desirable trait in a probiotic bacterium [14];
4. the selected strains should not carry transmissible antibiotic resistance genes.

### 2.2. Functional Criteria

For the selection of a probiotic strain, several criteria of functionality have to be considered. The functional criteria of probiotics should be established based on both *in vitro* and *in vivo* assays, and the results should be also reflected in controlled human studies. To deliver the health benefits, probiotics should be able to survive the acidic conditions of the upper GI tract and proliferate in the intestine, a requirement that is not always fulfilled. Feeding trials of Wistar rats with fermented milk containing free or immobilized *L. casei* ATCC 393 showed that the levels of the probiotic strain at the faeces and in the intestinal tissue dropped sharply and were undetected 48 h after discontinuation of administration [15, 16]. Apparently, daily consumption of probiotic products is a prerequisite for retaining cell levels at an effective concentration, information that could be valuable in the food industry. The continued existence of the probiotic strain in the human GI-tract has therefore been considered an essential trait. 

The survival of different probiotic strains in different parts of the GI-tract may greatly vary. Some strains are rapidly killed in the stomach, while others are able to pass through the whole gut in high numbers [17]. Bifidobacteria differ significantly in their survival in gastric juices [18–20] and bile salts [21, 22], as well as in their ability to adhere to epithelial cells [23, 24]. Moreover, because viable and biologically active microorganisms are usually required at the target site in the host, it is essential that probiotics are able to withstand the host's natural barriers against ingested bacteria. Several studies have shown that many strains of *Bifidobacterium* sp. intrinsically lack the ability to survive the harsh conditions of acidity and bile concentration commonly encountered in the GI-tract of humans [19, 25].

The reduction of viable cell levels might not always constitute a major issue, as a high number of studies reporting that nonviable probiotics could also have beneficial effects on human health or even be more efficient than alive cells are available [27, 28]. For example, lyophilized heat-killed *Lactobacillus acidophilus* was more effective than living lactobacilli in the treatment of chronic diarrhea [29]. Likewise, in the case of lactose tolerance by lactase-deficient subjects, viable and non-viable cultured milks show similar effects [27]. Similarly, in the treatment of acute gastroenteritis, some probiotics showed clinical efficacy in shortening the duration of diarrhoea both in viable and non-viable forms [27]. 

On the other hand, maintenance of cell viability is an essential requirement for the prevention and/or treatment of many disorders; that is, a daily dose of at least 10^8^ cells was required to restore and maintain a normal urogenital flora in women [30], supplementation of infant formulas with viable but not heat-inactivated LGG was proposed as a potential approach for the management of atopic eczema and cow's milk allergy [31], *Saccharomyces boulardii* was required in a viable form for the treatment of candidiasis, in contrast to lactic acid bacteria which showed efficacy both in the viable and non-viable forms [27], stimulation of the human immune system by oral administration of fermented milks or probiotic cultures has been observed with viable bacteria only [27], effects in faecal bacterial enzyme activities are observed following the consumption of viable bacteria only [27], and so forth. Hence, the association of viable cell levels with the clinical outcome is still dubious and seems to depend on the microbial species and on the disorder. Future work should focus on controlled blinded studies to further clarify issues concerning viability of probiotics during product manufacture and in the host, as well as to set the essential dosage for each case.

The health benefits of potential probiotic strains should be also assessed. Potential benefits may vary from maintenance of normal intestinal flora [15] to anticancer effects [32]. However, the positive activity might be strain specific and may be affected by the food matrix. Long-term clinical studies employing both animal models and humans are thus required to get fully proven health effects, especially to healthy populations.

### 2.3. Technological Criteria

Even though a probiotic strain fulfills the necessary safety and functional criteria, its selection should also satisfy technological criteria, as aspects related to probiotic food production and processing are also very important.

Viability of bacteria is often reduced during the food manufacture, distribution, and storage. Non-viable cultured products usually have longer shelf-life and easier storage which favour the adoption of the technology by the industrial sector, but it has been claimed that only probiotic products with viable microorganisms have beneficial health effects.

As it is strongly suggested that probiotic products should contain an adequate amount of live bacteria (at least 10^6^–10^7^ cfu/g) [33], the food industry has adopted the recommended level of 10^6^ cfu/g of probiotic cells at the time of consumption. Thus, a daily intake of at least 10^8^–10^9^ viable cells, which could be achieved with a daily consumption of at least 100 g of probiotic food, has been suggested as the minimum intake to provide a probiotic effect. Apart from high survival rates, the probiotic cultures should also not have a detrimental effect on sensory characteristics, for example, provide unpleasant flavours or textures.

Many surveys have shown large fluctuations and poor viability of probiotic bacteria, and especially bifidobacteria, in food products, such as yoghurt preparations [34, 35]. Several factors have been claimed to affect the viability of *Bifidobacterium* cultures in fermented milk products, including acidity [36], pH [37], concentration of lactic and acetic acids [38], hydrogen peroxide [39], and dissolved oxygen content [40]. The sensitivity of bifidobacteria to low pH and hydrogen peroxide combined with low viability in dairy products during storage at refrigeration temperature remains a problem in most fermented products [19, 39, 41]. Consequently, industrial demand for technologies ensuring bifidobacteria stability in foods is still strong, since high cell survival is important for both economical (a lower cell addition in the product is necessary when stability is high) and health effects, as well as business ethics issues (industry should not mislead the consumer by mentioning only the presence of probiotics without clarifying the amount of live bacteria at the time of consumption). Bifidobacteria are also very sensitive to environmental parameters and require expensive media for propagation and the addition of growth-promoting factors, due to their stringent growth requirements [42]. They are mainly marketed through fermented dairy foods, which are well suited to promote the health image of probiotics for several reasons. Firstly, fermented foods, and dairy products in particular, already have a positive health image, and consumers are familiar with the fact that these products contain living microorganisms [43]. Secondly, the image of yoghurt-like products as healthy foods facilitates the recommendation of daily consumption of bifidobacteria. Moreover, bifidobacteria have no adverse effects on the taste or aroma of dairy products and do not enhance acidification during product-shelf life [44]. Lastly, bifidobacteria are protected by milk proteins during digestion, which allows better delivery to the host [45]. 

As with all fermented dairy products containing living bacteria, products containing bifidobacteria must be cooled during storage, which is necessary both to guarantee high survival rates and to ensure product stability [34]. 

## 3. Immobilization and Encapsulation Technologies

The terms immobilization and encapsulation have been used interchangeably. Immobilization refers to the trapping of a material within or throughout a matrix, while encapsulation is the process of forming a continuous coating around an inner matrix that is wholly contained within the capsule wall as a core of encapsulated material. In both cases, the bidirectional diffusion of molecules, such as the influx of oxygen, nutrients, and growth factors, essential for cell metabolism and the outward diffusion of waste products should be permitted.

### 3.1. Immobilization and Encapsulation Techniques

Immobilization techniques often mimic nature, as naturally many microorganisms own the ability to adhere to and survive on different kinds of surfaces, and thus cells may grow within natural structures. 

**Figure 1:**
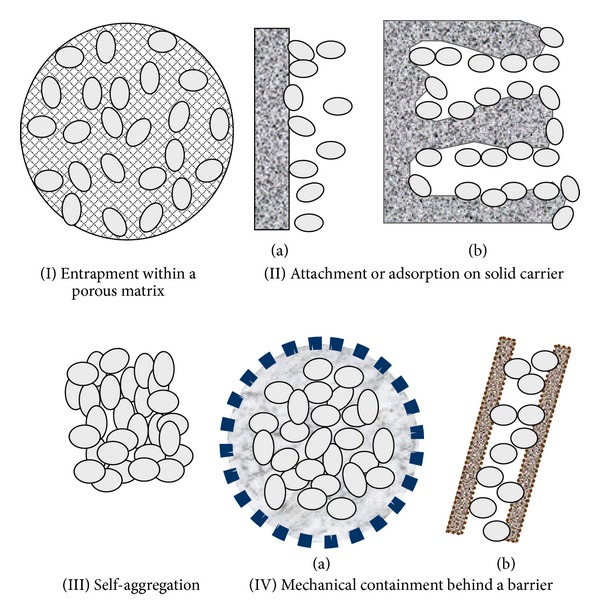
Basic methods of cell immobilization [26].

The immobilization methods can be divided into the following four major categories based on the physical mechanism employed (Figure 1) [26]:

1. entrapment within a porous matrix due to cells penetration until their mobility is obstructed by the presence of other cells or to formation of porous material *in situ* into a cell culture,
2. attachment or adsorption on solid carrier surfaces by physical adsorption due to electrostatic forces or by covalent binding between the cell membrane and the carrier,
3. self-aggregation by flocculation (natural) or with artificially induced cross-linking agents,
4. mechanical containment behind a barrier which could be either a microporous membrane or a microcapsule.

However, not all carriers are suitable for food production. Material used as a carrier should (a) have chemical, physical, and biological stability during processing and in the reaction conditions, (b) have sufficient mechanical strength, especially for its utilization in reactors and industry, (c) be nontoxic both for the immobilized cell and for the product, and (d) have high loading capacity. Material availability and cost-effectiveness of the immobilization process always have to be considered. Other criteria, such as physical characteristics (porosity, swelling, compression, and mean particle behavior), as well as possibility for microbial growth, biodegradability, and solubility, are application specific and should be also taken into account.  Table 4 summarizes the main prerequisites concerning cell immobilization supports.

### 3.2. Advantages of Cell Immobilization

Recent examples of research and applications on cell immobilization have emerged a series of advantages, which are summarized in Table 4. In brief, immobilisation of cells provides protection of cells agent against physicochemical changes, such as pH, temperature, bile salts [15, 73–76], higher cell densities and cell loads [77], higher productivity and efficiency [61], improved substrate utilization [61], reduced risk for microbial contamination [61], and faster fermentation and maturation rates, that is, in probiotic fermented milk and meat fermentation [61, 78].

## 4. Application of Immobilization Technology in Probiotic Food Production

Foods used for dissemination of probiotics are usually fermented foods even if probiotics could also be present in infant formulas, fruit drinks, whey drinks, and sweet milk. Fermented milk and cheese are the most common foods containing probiotics [33], while drinking and frozen yoghurts [79], ice creams [80], and fermented soya products [81] are well established in the market [82]. In fermented dairy products, most commonly lactobacilli such as *Lactobacillus acidophilus* and bifidobacteria, often referred to as “bifidus,” [83] are used as probiotics. Although strains of *Bifidobacterium* and *Lactobacillus* are currently the most widely used probiotics for human consumption, other microorganisms including *Enterococcus*, *Streptococcus*, and *Propionibacterium*, as well as some yeasts might also promote human intestinal health [84]. 

In Europe, probiotic applications are restricted to fermented milk products. In the United States, however, probiotics are found most frequently in the supplements sector and in yoghurts, while in Japan and Korea the use of probiotics is also widespread in food products that claim to assist the digestion process. On the other hand, research is also currently oriented to nondairy foods, like fermented meat and bakery products. 

Among the numerous immobilization supports, only a few are considered suitable for food production. For example, inorganic materials are usually excluded because they are characterized as unsuitable for human or animal nutrition. Instead, biopolymers and natural supports of food-grade purity are preferable. It is also very interesting to exploit materials with nondigestible carbohydrates and to investigate their application in probiotic food production.

Table 5 presents examples of the application of probiotic cell immobilization in food production. 

### 4.1. Microencapsulation on Biopolymers

Microencapsulation has been reported as a technology that can provide protection to sensitive cultures from high oxygen levels [85], manufacture and storage [86], freezing [87], and during transit through the human gastrointestinal tract [88]. 

#### 4.1.1. Alginates

Alginates are naturally derived linear copolymers of 1,4-linked *β*-D-mannuronic acid (M) and *α*-L-guluronic acid (G) residues. Aqueous solutions of polysaccharides form hydrogels in the presence of Ca^2+^ ions, resulting in physically cross-linked polymers with mechanical properties dependent on the alginate composition, as there is no regular repeat in alginate polymers (the chains can be described as varying sequence of regions of M, G, or MG blocks) [89].

Calcium alginate microspheres can be produced by both extrusion and emulsion techniques [77, 90–93]. 

Extrusion is the oldest and the most common approach to make capsules with hydrocolloids and might be achieved by simply dropping an aqueous solution of probiotics into a gelling bath. The size and shape of the beads usually range 2–5 mm and depend on the diameter of the needle and the distance of free fall [90]. It offers a small range size (smaller than emulsion), but it does not provide particles under 300 *μ*m [92]. Extrusion is more popular than emulsion technology due to its simplicity, easy handling, low cost at least to small scale, and gentle formulation conditions, which ensure maintenance of high cell viability (80–95%) [90]. Application of jet cutter technology allows today large-scale production of the microbeads [94].

In the emulsion technique, a small volume of cell-polymer suspension is added to a large volume of oil, and the mixture is homogenized to form a water-in-oil emulsion. Very often, the pH is reduced by addition of an oil-soluble acid, for example, acetic acid, enabling initiation of gelation with Ca^2+^. The size of beads depends on the speed of agitation and the type of emulsifier used. Therefore, it enables the production of the targeted microcapsules size [65], which can vary between 25 *μ*m and 2 mm [90]. The obtained capsules have a small diameter, but the main disadvantage of this method is that it provides large size range and shape particles. Due to the need for a vegetable oil, the operating cost may be higher than that of the extrusion technique [90]. The emulsion technique is relatively new to the food industry and easy to scale up for large-scale production and results in a high cell survival rate (80–95%) [90, 95]. 

**Figure 2:**
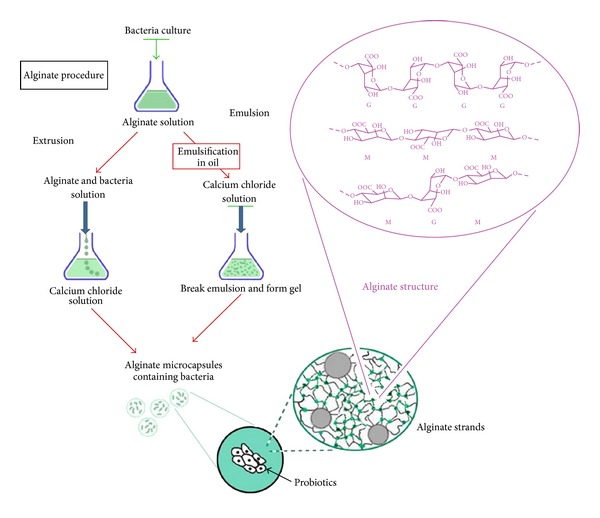
Steps of extrusion and emulsion processes and the chemical structure of the alginate residues along with a schematic diagram of the microorganisms and the hydrogels.

Figure 2 presents the steps of extrusion and emulsion processes and the chemical structure of the alginate residues along with a schematic diagram of the microorganisms and the hydrogels. 

An important challenge for probiotic encapsulation is to reduce the particle size, because it can negatively affect the textural and the sensorial properties of the product. For example, consumers detected a grainy texture in yogurts containing encapsulated bifidobacteria (size range particles about 22–50 *μ*m) [96]. On the other hand, there are already commercial products available (a yoghurt and a breakfast cereal) where particles containing probiotics are clearly seen in the foods and even advertised on the label. With gel particles, the cells are typically not released into the food products when added; *in vitro* and *ex vivo* studies showed that beads maintained their integrity in simulated stomach conditions but subsequently released their cargo in the GI tract [91].

Another issue that should be addressed is that the presence of residual oil on capsule surface produced by emulsification is detrimental to the texture and the organoleptic properties of the product. Also, capsules incorporation in diet products is hampered, and the residual oil, surfactant, or emulsifier can be toxic for probiotic cells.

The survival of the microencapsulated probiotics*, Lactobacillus acidophilus* 547, *Bifidobacterium bifidum* ATCC 1994 and *Lactobacillus casei* 01, in stirred yoghurt from Ultrahigh temperature (UHT)—and conventionally treated milk during low temperature storage—was investigated by Krasaekoopt et al. [63]. Higher survival of encapsulated probiotic bacteria in alginate beads coated with chitosan compared to free cells by approximately 1 log cycle was recorded. The number of probiotic bacteria remained above the recommended therapeutic minimum (10^7^ cfu/g) throughout the storage period (4 weeks), except for *B. bifidum*, the levels of which decreased below 10^6^ cfu/g (37.6–47.5% reduction of viable cell counts). The use of UHT or conventionally treated milks had no effect on cell survival. As bifidobacteria are strictly anaerobic, the survival of this organism may be improved by increasing the initial number of cells before encapsulation and by addition of an oxygen scavenger, such as L-cysteine hydrochloride, during microencapsulation [63]. 

Microencapsulation also appeared to create anoxic regions inside the microcapsules, therefore reducing oxygen, which prevented viability losses of oxygen-sensitive probiotic strains, in addition to protecting the cells against the acid conditions in yoghurt [97, 98]. Oxygen tolerance of bifidobacteria in gel beads was also further confirmed [99]. However, the efficiency of microencapsulation in protecting probiotics depends on the oxygen sensitivity of the strain and the dissolved oxygen levels in the food product.

Ca-alginate entrapment of *Lactobacillus acidophilus* was also proposed for the production of probiotic fermented tomato juice [68]. The immobilized cells endured the adverse effects of tomato juice, and thus the viable counts of immobilized cells were maintained in levels ≥7 log cfu/g after 10 weeks of cold storage at 4°C, in contrast to 4 log cfu/g of free cells.

Similarly, survival of calcium-induced alginate-starch encapsulated *L. acidophilus* and *B. lactis* was significantly improved when inserted in yoghurt, due to protection of cells [65]. The same trend was also reported during storage of freeze-dried yoghurt in ambient temperature containing alginate-microencapsulated mixed probiotic culture [100] and in alginate-microencapsulated *L. reuteri* produced using either extrusion or emulsion technology incorporated in dry-fermented sausages [66], as well as in calcium alginate encapsulated *B. bifidum* and *B. infantis* inserted in mayonnaise [60]. 

A symbiotic pastry product was previously prepared by incorporating free or encapsulated *Lactobacillus casei* NCDC 298 in sodium alginate in milk chocolate together with inulin [71]. Although cell encapsulation resulted in significant increase of cell survival at low pH, high bile salt concentration, and during heat treatment [101], the viable counts of both free and encapsulated *L. casei* NCDC 298 were unchanged during the storage of milk chocolate at refrigerated conditions up to 60 days and were higher than the recommended level by International Dairy Federation guidelines (10^7^ cfu/g) at the end of the product shelf-life [71]. Feeding of the symbiotic chocolate increased the fecal lactobacilli and decreased fecal coliforms and **β**-glucuronidase activity in mice, indicating that it might constitute an excellent food for delivery of probiotic lactobacilli [71].

On the contrary, encapsulation of *Lactobacillus acidophilus* ATCC 4356 on calcium alginates had no effect on cell survival compared to free cells during refrigerated storage of yoghurts for 4 weeks [70]. However, significantly greater survival of encapsulated over free probiotic bacteria was observed in the *in vitro* assays using artificial human gastric digestion systems [70]. 

In another study, it was reported that microencapsulation in alginates resulted in enhanced resistance of *L. casei* to heat processing at 55–65°C [101]. The data suggested that microencapsulated probiotic cells could be used in meat processing, which require moderate heat treatments.

#### 4.1.2. Milk and Whey Protein Gels

Encapsulation of probiotics in whey protein gel particles may offer protection during food processing and storage. The protein microcapsules containing the encapsulated bacteria can be produced by emulsion and spray drying or extrusion and freeze drying and they may be incorporated in various products, such as yoghurt, cheese, and biscuits, to confer probiotic properties [67, 102–105]. Immobilization of probiotic cultures in whey protein-based microcapsules can increase cell survival when subjected to extreme conditions, making this approach potentially useful for delivery of viable bacteria to the gastrointestinal tract of humans via dairy fermented products. However, technological properties of the strains, and particularly heat resistance, should be taken into consideration for spray-drying encapsulation of sensitive microorganisms.

*Lactobacillus paracasei* ssp. *paracasei* F19 and *Bifidobacterium lactis* Bb12 were encapsulated in milk protein matrices by means of an enzymatic induced gelation with rennet [74] and in food grade casein microcapsules based on a transglutaminase-catalysed gelation of casein suspensions [75]. Water insoluble, spherical capsules with a volume-based median diameter of 68 ± 5 mm and 165 ± 23 mm were obtained, respectively. Analysis of living cell numbers after incubation of free and encapsulated probiotics at low pH values and in simulated gastric juice without pepsin at pH 2.5 and pH 3.6 (37°C, 90 min) showed a protective effect due to microencapsulation under all conditions tested. Both studies indicated that the microencapsulation of probiotic cells can be a suitable alternative to current available technologies and can protect probiotic cells from damage due to pH levels similar to those in the human stomach.

The efficacy of whey protein isolate as an encapsulation matrix for the maintenance of *Lactobacillus rhamnosus* GG viability was previously evaluated [106]. Atomic force microscopy demonstrated that microbead extrusion at pH 4.6 stimulated strong cohesive interactions within protein-probiotic amalgams. Live/dead microscopy staining visualized the homogenous distribution of live probiotics throughout micro-bead matrices. Following 3 h *in vitro* stomach incubation (pH 1.8; 37°C), micro-beads laden with 10^10^ cfu demonstrated acid stability and peptic resistance, characteristics required for optimum probiotic refuge. However, enzyme-activated intestinal conditions catalysed a synergistic response engaging rapid matrix disintegration and controlled probiotic release. Overall, the study led to the development and design of a protein encapsulation polymer based on congruent matrix interactions for reinforced probiotic protection during challenging situations for their targeted delivery to intestinal adsorption sites.

Accordingly, cell immobilization of the same strain (*Lactobacillus rhamnosus* GG) in native, denatured, and hydrolysed whey protein isolates was investigated by Doherty et al. [76]. Hydrolysed or denatured whey protein isolates were the most suitable matrices for cell immobilization, while native protein provided the weakest safeguard against thermal and acid stress. Spatial distribution of probiotic cells within immobilized treatments was evaluated by atomic force and confocal scanning laser microscopy and microscopic analysis of denatured treatments revealed an oasis of immobilized cells, phase separated from the surrounding protein matrix.

Direct dispersion of fresh cells in a heat-treated whey protein suspension followed by spray drying was also proposed as an alternative and less destructive microencapsulation method, with survival rates after spray drying of 26% for *B. breve* and 1.4% for the more heat-sensitive *B. longum* [102]. Even though the viability of the bacteria after spray drying remained low, viable counts of *B. breve* cells entrapped in whey protein microcapsules were significantly higher than those of free cells after 28 days in yoghurt stored at 4°C (+2.6 log cycles) and after sequential exposure to simulated gastric and intestinal juices (+2.7 log cycles). In contrast, no protective effect of encapsulation was observed with *B. longum*.

Likewise, the effect of whey protein isolate gel microentrapment on the viability of *Lactobacillus rhamnosus* R011 during the production and storage of biscuits, frozen cranberry juice, and vegetable juice was investigated by Ainsley Reid et al. [67]. The beads were produced by extrusion of the denatured whey protein isolate-concentrated bacteria solution (70 : 30 volume ratio) in a CaCl_2_ solution. After soaking for 30 min at 4°C in a sterile milk-based protective solution consisting of 20% (w/w) skim milk powder, 5% (w/w) sucrose, 1% (w/w) bacto casitone, and supplemented with 0.35% (w/v) ascorbic acid, they were freeze dried and mashed to obtain a fine powder [67]. Viability of microentrapped cells was compared to free cells freeze-dried in the milk-based protective solution and in a denatured whey protein isolate-based solution (ungelled) enriched with lactose and sucrose. During the production of biscuits and their storage for 2 weeks at 23°C, the minimum drop in cell counts (from 1.3 × 10^7^ to <10^3^ cfu/g) was observed in cells microentrapped in whey protein isolate gel particles. However, free cells prepared in the milk-based matrix maintained the highest viability during storage of vegetable juice, as well as during freezing and storage of cranberry juice. The highest reduction in viable counts during the heating process of biscuits, as well as during storage of vegetable juice and freezing and storage of cranberry juice, was recorded in the free culture prepared in the whey protein isolate-based solution. Although the whey protein isolate-based solution was not efficient in maintaining high viability of free cells, it was concluded that the process of microentrapment may help in protecting the freeze-dried cells against subsequent acidic and alkaline pH conditions and during the heating and freezing of food products.

### 4.2. Natural Supports

Fruits contain non-digestible carbohydrates, which constitute the base for cell immobilization. Apple and quince pieces proved to be suitable supports for immobilization of *Lactobacillus casei* cells [61]. The immobilized biocatalysts were used in lactic acid and probiotic additive fermented milk production, while the immobilized bacterial cells were able to reactivate after storage for 129 days at 4°C. In the fermented milk, a fruity, distinctive aroma was predominant during all the storage period. Immobilized *L. casei* cells on fruit pieces have also been successfully used in probiotic cheese production [62]. 

Fruit and oat pieces were also recently proposed as vehicles for delivery of *L. casei* ATCC 393. The immobilized cells were used for probiotic yoghurt production, and cell survival was monitored during refrigerated storage. Microbiological and strain-specific multiplex PCR analysis showed that both free and immobilized *L. casei* ATCC 393 were detected at necessary levels for conferring a probiotic effect (at least 6 log cfu/g) for longer periods than required by the dairy industry (≥30 d) during storage at 4°C [72].

Attempts were carried out to combine the beneficial effects of probiotics with fruit and vegetables by applying the vacuum impregnation process. It was shown that it is possible to introduce microbial cells into structural matrix of fresh apple tissue by using impregnation liquid inoculated with *S. cerevisiae* and *L. casei* spp. *rhamnosus*. A process combining vacuum impregnation process with air drying was proposed for developing dried fruit products with probiotic effects [107]. Vacuum and/or atmospheric impregnation techniques are considered as feasible technologies for exploitations of fruit and vegetable tissues. Functional ingredients can be successfully incorporated into plant-origin tissues providing, thus, novel functional product categories and new commercial opportunities.

The aim of preservation techniques for foods of concern in “Ibero-America” (CYTED Program), carried out from 1999 to 2004, was to analyze the feasibility of atmospheric and/or vacuum impregnation treatments in order to incorporate physiologically active compounds into plant tissues without destroying the initial food matrix. The above research contributed significantly in the development of functional fruit and vegetable matrices enriched with probiotics [108].

Cereals, which also contain non-digestible carbohydrates, could be applied as supports for cell immobilization. During the last years, several encapsulation techniques using cereal fractions have been tested in order to improve the viability of the probiotic strains in functional foods [109, 110]. The multiple beneficial effects of cereals can be exploited in different ways leading to the design of novel foods. Cereals can be used as fermentable substrates for the growth of probiotic microorganisms, but they also contain potential prebiotic compounds, the functional properties of which should be explored. In addition, cereal constituents, such as starch, are expected to have the ability to deliver immobilized probiotic microorganisms to the human gut when used as immobilization supports [111, 112]. 

Based on the above perspective, wheat dextrin, polydextrose, apple fibre, and inulin were considered promising carriers of *Lactobacillus rhamnosus* during freeze drying and storage in apple juice and chocolate coated breakfast cereals [64].

Recently, production of probiotic dry fermented sausages containing immobilized *Lactobacillus casei* ATCC 393 on wheat was assessed (unpublished results). The levels of the probiotic strain remained higher than 6 log cfu/g during the ripening process but more importantly after heat treatment at 70–72°C for 8–10 min in contrast to free cells, confirming the protective role of cell immobilization.

Finally, efforts to immobilize probiotic strain on agricultural wastes were recently carried out. Rinds of durian, mangosteen, and jackfruit were used as supports for immobilizing strains of *L. acidophilus* and *L. bulgaricus,* and the immobilized probiotics showed increased growth, greater reduction of stachyose, sucrose, and glucose, higher production of lactic and acetic acids, and lower pH in soy milk compared to free cells [69]. Similarly, *L. casei* was immobilized on brewer's spent grains, and the immobilized biocatalyst was used in bread making [113]. However, the products were not characterized as probiotics, since *L. casei* cells did not survive the baking process. 

## 5. Conclusions and Future Perspectives

Despite the plethora of probiotic products and the immobilization supports proposed by several researchers, the immobilized cell technology has not yet been widely adopted by the industrial sector, mainly due to safety issues related to the immobilization agents, confirmation of the stability, and functionality of bioactive cultures and the lack of processes that can be readily scaledup. Ongoing research aims at resolving the above issues, as immobilization is a successful way of protecting and improving cell viability. The assessment of the industrial feasibility of immobilization technology is mandatory for providing cost-effective, large-scale quantities of probiotic products for specific clinical and/or commercial use. 

The crucial factors for the implementation on an industrial level are carrier materials, immobilization technology, and bioreactor design. Although research on immobilized cells has been carried out for several years, many difficulties related to the application at industrial scale still exist. The two most important disadvantages that should be always kept on mind are complexity of production process and cost limitations. At the moment, the major challenges for successful application of immobilized cell technology in industrial probiotic food processing are evidence of enhancement of cell viability, effective clinical outcome versus free cells and finetuning of the organoleptic characteristics. In fact, engineering problems linked to choice of the carrier and reactor design are complicated by the safety of immobilizing agents and the effects of immobilization on the sensory attributes of the final products. Future research should be focused on overcoming the gap between conditions at research level and demands for large-scale applications, improving existing manufacturing technologies, and choosing new processing conditions and new carrier materials. Furthermore, future studies should be oriented to preservation and storage techniques that could be easily adopted at the industrial level.

For successful immobilization and cultivation of probiotic cells, the immobilization material must be conducive to cell viability and function (biocompatible) within specific food systems. Hence, immobilization supports of food grade purity, such as natural supports, are considered advantageous for food production. Microcapsule or bead systems using various biopolymers are very easy to prepare on a lab scale. However, the scaling up of the process is very difficult, and processing costs are very high. In addition, mechanical instability is an important disadvantage of gels. It has been often noticed that the gel structure is being destroyed due to cell growth and intensive carbon dioxide production. 

In the near future, multiple deliveries are expected to be the key factor, and thus a new area of complex nutritional matrices will be augmented. For example, coencapsulation of probiotic cultures with certain food ingredients may be beneficial, as at the same time it enables introduction of bioactive compounds, while the positive effects of probiotics can be enhanced with the right selection of substances. Hence, coimmobilization of probiotic microorganism with prebiotics, antioxidants, peptides, or immune-enhancing compounds is becoming especially attractive in future perspectives. 

A number of efficient materials and the associated controlled release mechanisms are currently under investigation. It is expected that new innovative ways of administration and delivering of probiotics will be developed shortly. However, more research is still required for the selection of immobilization supports that can trigger successful adhesion to specific intestinal cells, therefore achieving targeted delivery of probiotic bacteria to various sites within the GI tract. More *in vivo* studies should be conducted using human subjects to confirm the efficacy of micro- or nanoencapsulation in transporting probiotic bacteria and their controlled release in the GI system. Additional evidence based on clinical data is still required on the safety of the immobilization supports and on the effect of immobilization on the effectiveness of the probiotics in comparison to free cells.

Additionally, new food regulation should specify labelling including the strain and the number of viable cells at the end of the shelf-life of probiotic-claimed foods. Such directives are considered crucial for the development of industrial and commercial consciousness and for the consumer protection. 

Finally, the development of novel functional foods is a major challenge to address the expectation of consumers for healthy and beneficial food products. Industries should overcome the possible difficulties and find ways to exploit the advantages offered by the immobilized cell technology with an adequate cost. It is evident that the probiotic market has a strong future, as the benefits provided by probiotics consumption are now well documented, and thus consumer requirements are expected to increase.

## Figures and Tables

**Table 1:** Most common microorganisms studied for probiotic properties.

Lactobacilli	
*L. acidophilus *	
*L. casei *	
*L. rhamnosus *	
*L. reuteri *	
*L. plantarum *	
*L. fermentum *	
*L. johnsonii *	
*L. helveticus *	
*L. farciminis *	
*L. curvatus *	
*L. brevis *	
*L. gasseri *	
*L. salivarius *	
*L. cellobiosus *	
Bifidobacteria	
* B. bifidum *	
* B. breve *	
* B. infantis *	
* B. longum *	
* B. lactis *	
* B. thermophilum *	
* B. adolescents *	
* B. animalis *	
Other bacteria	
* Enterococcus faecium *	
* Escherichia coli *Nissle 1917	
* Lactococcus lactis *	
* Propionibacterium freudenreichii *	
* Bacillus clausii *	
* Bacillus oligonitrophilis *	
Yeast	
* Saccharomyces boulardii *	
* Saccharomyces cerevisiae *	

**Table 2:** Most important beneficial effects of probiotics.

	Beneficial effect	Probiotic microorganism	Type of trial	Outcome	Reference
Metabolism	Lactose digestion	*Lactobacillus casei* Shirota	Target group of patients, lactose maldigestion	*Lactobacillus casei* Shirota seems to improve symptoms in lactose-intolerant patients	[46]
	Lipid metabolism	*Lactobacillus plantarum*, *Lactobacillus curvatus *	Randomized	*Lactobacillus plantarum* and *Lactobacillus curvatus* reduced cholesterol in plasma and liver	[47]
	Oxalate metabolism	*Lactobacillus casei *	Target group of patients, stone-forming patients	*Lactobacillus casei* processed lowering effect upon urinary oxalate excretion	[48]

Chronic intestinal inflammatory and functional disorders	Inflammatory bowel diseases (IBD): Crohn's disease	*Saccharomyces boulardii *	Pilot study	*Saccharomyces boulardii* controlled inflammation and promoted epithelial restitution	[49]
	Ulcerative colitis	Combined lactobacilli and enterococci	Randomized	Effective treatment by mediating immunological response	[50]
	Irritable bowel syndrome (IBS)	*Lactobacillus plantarum *	Double blind, placebo controlled, and parallel designed	*Lactobacillus plantarum* provided effective symptom relief	[51]

Allergic diseases	Eczema	*Lactobacillus rhamnosus *	Randomized, double blind, placebo controlled	*Lactobacillus rhamnosus* showed protective effect against eczema	[52]
	Atopic dermatitis	*Lactobacillus plantarum *	Randomized, double blind, placebo controlled	*Lactobacillus plantarum* was beneficial in the treatment of pediatric AD	[53]
	Allergic rhinitis	*Lactobacillus salivarius *	Randomized, double blind controlled	*Lactobacillus salivarius* reduced symptoms and drug usage in children	[54]
	Asthma	*Lactobacillus gasseri *	Randomized, placebo controlled	*Lactobacillus gasseri* had clinical benefits in asthma treatment	[55]

Reduction of risk factors of infection	Infectious diarrhea	*Lactobacillus acidophilus*, *Lactobacillus rhamnosus*, *Bifidobacterium bifidum*, and *Bifidobacterium longum *	Randomized, single blind	Synbiotic mixture showed reduction in diarrhea duration	[56]
	Necrotizing enterocolitis (infants)	*Lactobacillus acidophilus*, *Bifidobacterium infantis *	Randomized, placebo controlled	Protective effect in clinical NEC	[57]
	*Helicobacter pylori *	*Lactobacillus acidophilus*, *Lactobacillus rhamnosus*, *Lactobacillus bulgaricus*, *Lactobacillus casei, Streptococcus thermophilus, *and* Bifidobacterium infantis Bifidobacterium breve *	Randomized, double blind, placebo controlled	Positive effect on the eradication of *H. pylori* infection	[58]

Respiratory tract infections	Ear, nose, and throat infections	*Lactobacillus rhamnosus* GG	Randomized, double blind, and placebo controlled	*Lactobacillus rhamnosus* GG reduced the risk of early acute otitis, the antibiotic use, and the risk of recurrent respiratory infections during first year of life	[59]

Malignancy	Cervical cancer	*Bifidobacterium adolescentis *	Prospective controlled pilot study	Probiotic studies promoted the clearance of HPV-related cytological abnormalities	[32]

**Table 3:** Criteria used to define a probiotic microorganism.

Safety criteria	Be of human origin
	Nonpathogenic in nature
	Generally recognized as safe (GRAS)

Functional criteria	Be resistant to destruction by gastric acid and bile salts
	Adhere to intestinal epithelial tissue
	Be able to colonize the gastrointestinal tract, even in the short term
	Modulate immune responses
	Produce antimicrobial substances
	Influence human metabolic activities (i.e., cholesterol assimilation, lactase activity, vitamin production, etc.)

Technological criteria	Be resistant to destruction by technical processing
	Be subjected to scale-up processes

**Table 4:** Prerequisites of immobilization supports and advantages of cell immobilization.

Prerequisites of immobilization supports	Advantages of cell immobilization
(1) Adequately large surface of the immobilization support (2) Easy handling and regeneration of the immobilization support (3) Availability of the immobilization support (4) Cost-effectiveness of the support and immobilization process (5) Acceptance of immobilization support by the consumers and avoidance of negative effects on the final food product (e.g., off-flavour formations) (6) Retention of immobilized cell viability (7) Avoidance of negative effects of cell immobilization on biological and metabolic activity of immobilized cells (8) Food-grade purity of the immobilization support	(1) Prolonged activity and stability of the immobilized cells, since the immobilization support may act as a protective agent against physicochemical changes (pH, temperature, bile salts, etc.) (2) Higher cell densities which lead to higher productivities and increased substrate uptake and yield (3) Increased tolerance to high substrate concentration and final product inhibition (4) Reduction of risk of microbial contamination due to high cell densities and enhanced fermentation activity (5) Ability for low-temperature fermentation and/or maturation for certain food products (6) Reduction of fermentation and maturation times in certain circumstances

**Table 5:** Characteristic examples of application of probiotic cell immobilization in food production.

Immobilization/encapsulation support	Probiotic microorganism	Probiotic food product	Reference
Alginate encapsulation	*Bifidobacterium bifidum, Bifidobacterium infantis *	Mayonnaise	[60]

Apple pieces, quince pieces	*Lactobacillus casei * ATCC 393	Fermented milk	[61]

Apple, pear pieces	*Lactobacillus casei * ATCC 393	Cheese	[62]

Chitosan coated alginate beads	*Lactobacillus casei * 01, *Lactobacillus acidophilus * 547	Yogurt	[63]

Fibres	*Lactobacillus rhamnosus* E800, E522 *Lactobacillus acidophilus * DD910	Apple juice, chocolate coated breakfast cereals	[64]

Calcium induced, encapsulated alginate starch	*Bifidobacterium lactis * DD920	Yogurt	[65]

Microencapsulation in alginate	*Lactobacillus reuteri *	Sausages	[66]

Whey protein	*Lactobacillus t* *rhamnosus* R011	Biscuits, frozen cranberry juice, and vegetable juice	[67]

Calcium alginate	*Lactobacillus acidophilus* BCRC 10695	Tomato juice	[68]

Rinds of durian, mangosteen, and jackfruit	*Lactobacillus acidophilus* FTDC 1331, 2631, 2333, and 1733 and *Lactobacillus bulgaricus* FTDC 0411	Soy milk	[69]

Calcium alginate	*Lactobacillus acidophilus* ATCC 4356	Yogurt	[70]

Sodium alginate	*Lactobacillus casei* NCDC 298	Synbiotic milk chocolate	[71]

Fruits, oat pieces	*Lactobacillus casei* ATCC 393, *Lactobacillus delbrueckii* ssp. *bulgaricus *	Yogurt	[72]

Wheat grains	*Lactobacillus casei* ATCC 393	Fermented sausage	[unpublished results]
